# Research on the sustainable development of agricultural product supply chain in three northeast provinces in China

**DOI:** 10.3389/fpubh.2022.1007486

**Published:** 2023-01-06

**Authors:** Xuemei Fan, Yingdan Zhang, Yuanhang Ma, Cong Zhao, Buxin Liang, Hao Chu

**Affiliations:** School of Business and Management, Jilin University, Changchun, China

**Keywords:** sustainable development, agricultural product supply chain, entropy weight-matter-element extension model (MEEM), autoregressive integrated moving average model (ARIMA), three northeast provinces in China

## Abstract

**Background:**

The sustainable development of the agricultural product supply chain (APSC) is the key to protecting public health.

**Methods:**

This paper explores the sustainable development status of the APSC in three northeast provinces of China from 2007 to 2020 and the development trend in the next 5 years by using the entropy weight—matter-element extension model (MEEM) and autoregressive integrated moving average model (ARIMA), taking into account the background of relatively backward development and the high proportion of agricultural output in these three provinces.

**Results:**

According to the research results, the sustainable development of the APSC in Jilin Province is relatively stable, Heilongjiang Province has made considerable progress in the sustainable development of the APSC in recent years, while Liaoning Province has shown a significant downward trend in recent years in the sustainable development of the APSC, despite a strong development momentum in previous years.

**Conclusions:**

The findings of this paper can be applied to the governance of APSC in other rural areas with uneven development. The assessment also provides guidance on the quality and safety of agricultural products and public health, and raises the awareness of policymakers on the importance of the APSC.

## Introduction

Sustainable development is an urgent and important development strategy to address a myriad of challenges such as climate change and ecological crisis (1, 2). Sustainable development offers a feasible solution for global challenges such as poverty, public health, and climate change (3). Nowadays, both developed countries and developing countries, based on their consensus on sustainable development, have come to realize the urgency of setting sustainability goals and the need to incorporate them as part of the scientific and systematic decision-making strategy, which further proves the necessity of studying sustainable development in various fields. In particular, agriculture as the basic industry of each country is related to the national economy and social stability. Agricultural products are a basic human need (4).

The agricultural product supply chain (APSC) is an important means to ensure consumers have access to healthy fresh food. However, the process of turning agricultural products from raw materials into finished products involves a process that will impose a huge burden on the environment, which is part of the management of APSC (5). The sustainable development of the APSC exerts critical impacts in various areas, such as employment, income growth, market opportunities for consumers and producers, and existing businesses running through supply chains in many countries (6–8). Many scholars have found that a focus on the APSC can improve social, economic, and environmental impacts, improve product quality and public health, and lead to better sustainable development (9, 10).

Government support is required for the sustainable development of the APSC. On the basis that all APSC stakeholders should work together, government initiatives are the causal category with the greatest impact on the sustainability of APSC (11). Government subsidies are the main driver of agricultural supply chain sustainability (12). There is a need to increase the investment and management of infrastructure related to APSC to give full play to its leading role (13, 14). At the same time, it is necessary for the government to improve the policies and regulations related to APSC (15).

Through the collation of the literature, it can be concluded that most scholars have noticed that each process involved in the APSC will have an impact on the environment, society, and economy, which is contrary to the meaning of sustainable development. Meanwhile, some scholars mentioned the importance of government participation in management, which can effectively improve the potential of sustainable development. In terms of research methods, some scholars conducted micro-level evaluation and analysis by establishing measurement and stage models, and most of them used case and policy analysis to study from a macro perspective. Few research methods combine theory with practice. Combined with some scholars' analysis of China's agriculture, the three northeast provinces of China have a relatively well-established agricultural structure, rich land resources, and abundant agricultural output. However, the development model of APSC is relatively extensive, and there are not much relevant theoretical research and case analysis, especially the overall comparative analysis of the three provinces.

Therefore, combined with the principle of the barrel effect, it is imperative to systematically study the sustainable development of APSC in the three northeast provinces of China from a macro perspective, so as to offer inspiration for promoting rural revitalization in northeast China and the sustainable development of China's APSC. In addition, this paper adopts the MEEM to solve the contradictory problems both qualitatively and quantitatively, and then used the ARIMA model to make a short-term prediction, which can produce more systematic and scientific results, and will better combine theory and practice in evaluation and management. This paper also provides guidance for ensuring the health and life safety of residents, which is of great value for the sustainable development of APSC in regions with similar conditions in other countries.

## Methods and indexes

### MEEM model

The matter-element extension method is introduced by Cai Wen and other Chinese scholars to solve contradictory problems and it can also be applied to comprehensive evaluation research. The entropy weight—matter-element extension model (MEEM) can greatly reduce the influence of subjective factors in evaluations and combine described objects, characteristics and value into matter-elements for analysis (16–18).

1. Define the matter-element matrix to be evaluated

Matter-element is a unity of a set of described objects, their characteristics, and eigenvalue. The matter-element matrix is shown as follows:


(1) $$\begin{array}{l} \begin{array}{l} R=(M,C,V)=\left[\begin{array}{ccc} M & C_{1} & V_{1}\\ & C_{2} & V_{2}\\ & \vdots & \vdots \\ & C_{n} & V_{n} \end{array}\right] \end{array} \end{array}$$

where M is the described object, C is the characteristics of the object and V is the value.

2. Set up classical domain and joint domain matter-element

The classical domain mainly describes the range of eigenvalue changes of the described object:


(2) $$\begin{array}{l} R_{\text{j}}=(M_{j},C,V_{j})=\left[\begin{array}{ccc} M_{j} & C_{1} & \left(a_{j1},b_{j1}\right)\\ & C_{2} & \left(a_{j2},b_{j2}\right)\\ & \vdots & \vdots \\ & C_{n} & \left(a_{jn},b_{jn}\right) \end{array}\right] \end{array}$$

The joint domain:


(3) $$\begin{array}{l} R_{u}=(M_{u},C,V_{u})=\left[\begin{array}{ccc} M_{u} & C_{1} & \left(a_{1},b_{1}\right)\\ & C_{2} & \left(a_{2},b_{2}\right)\\ & \vdots & \vdots \\ & C_{n} & \left(a_{n},b_{n}\right) \end{array}\right] \end{array}$$

*R*_u_ indicates the collection of grades, *V*_*u*_ indicates the value range of the evaluation index, *a*_*n*_ indicates the lower bound of the evaluation index *C*_*n*_, and *b*_*n*_ indicates the upper bound.

Since most of the classical domains and joint domains are defined by norms or standards, this paper improves grade ranges with the time-series approach based on its data structure (19):

Time series of a certain index *C*_i_ is index is defined as grade J in the following steps:

1) Calculate the interval's length L of the time series

(4) $$\begin{array}{l} \text{L}=\max C_{i}-\min C_{i} \end{array}$$

2) Calculate the average interval G of the time series

(5) $$\begin{array}{l} G=\frac{L}{J}=\frac{\max C_{i}-\min C_{i}}{J} \end{array}$$

3) Calculate the range bounds of each grade. The difference between the upper and lower bounds of each grade is G and the lower bound of each grade equals the upper bound of its adjacent grade.

(6) $$\begin{array}{l} C_{\text{ij}}=\left[\min C_{i}+\left(j-1\right)G_{i},\min C_{i}+jG\right] \end{array}$$

3. Evaluate the matter-element correlation

The distance from *X*_i_ to the classical domain range *X*_ji_ and to the joint domain range *X*_ui_ is calculated as follows respectively:


(7) $$\rho ({\text{x}}_{\text{i}},X_{ji})=\left|x_{i}-\frac{1}{2}(a_{ji}+b_{ji})\right|-\frac{1}{2}(b_{ji}-a_{ji})$$

(8) $$\begin{array}{l} \rho ({\text{x}}_{\text{i}},X_{ui})=\left|x_{i}-\frac{1}{2}(a_{ui}+b_{ui})\right|-\frac{1}{2}(b_{ui}-a_{ui}) \end{array}$$

The correlation function *K*_j_(*X*_*n*_) is:


(9) $$K_{\text{j}}(X_{i})=\{\begin{array}{c} -\frac{\rho ({\text{x}}_{\text{i}},X_{ji})}{\left|X_{\text{ji}}\right|},x_{i}\in X_{ji}\\ \frac{\rho ({\text{x}}_{\text{i}},X_{ji})}{\rho (x_{i},X_{ui})-\rho ({\text{x}}_{\text{i}},X_{ji})},x_{i}\notin X_{ji} \end{array}$$

where |*X*_ji_| = |*b*_*ji*_ − *a*_*ji*_|

4. Determine the index weight

To ensure that index weights are scientific and effective and to minimize errors, this paper adopts the entropy value method to determine index weights. Entropy represents the measurement of uncertainty, in other words, it evaluates the index's disorder degree and reflects the differentiation degree of evaluated units. The higher the entropy value, the more disordered the sample; the less information it includes, the smaller the weight. In this method, each index's entropy value is firstly calculated according to the entropy function, then the entropy value is normalized into index weight. The specific steps are as follows (20, 21):

1) Normalize the index

(10) $$\begin{array}{l} {x_{ij}}^{+}=\frac{x_{ij}-\max\left\{x_{1j,......}x_{nj}\right\}}{\max\left\{x_{1j,......}x_{nj}\right\}-\min\left\{x_{1j,......}x_{nj}\right\}} \end{array}$$

(11) $$\begin{array}{l} {x_{ij}}^{-}=\frac{\max\left\{x_{1j,......}x_{nj}\right\}-{\text{x}}_{ij}}{\max\left\{x_{1j,......}x_{nj}\right\}-\min\left\{x_{1j,......}x_{nj}\right\}} \end{array}$$

where *x*_*ij*_ indicates the jth index in the ith year.

2) Calculate the entropy

(12) $$\begin{array}{l} {\text{e}}_{j}=-k\overset{n}{\underset{i-1}{\sum }}P_{ij}\ln\;\left(P_{ij}\right) \end{array}$$

Entropy is the total contribution of the selected year to the jth index and K is a constant:


(13) $$\begin{array}{l} k=\frac{1}{\ln\;n}\left(0\leq e_{j}\leq 1\right) \end{array}$$

(14) $$\begin{array}{l} P_{ij}\text{=}\frac{{\text{x}}_{ij}}{\overset{n}{\underset{i=1}{\sum }}{\text{x}}_{ij}} \end{array}$$

3) Calculate the index's difference coefficient

(15) $$\begin{array}{l} d_{j}=1-e_{j} \end{array}$$

4) Determine the weight index

(16) $$\begin{array}{l} \omega_{\text{j}}=\frac{{\text{d}}_{\text{j}}}{\overset{\text{m}}{\underset{\text{i}=\text{1}}{\sum }}{\text{d}}_{\text{j}}}\;,\;j=1,2,\cdots \;,m \end{array}$$

5) Calculate the comprehensive correlation degree of evaluated elements in each level

(17) $$\begin{array}{l} K_{\text{j}}\left(M_{\text{x}}\right)=\overset{\text{n}}{\underset{i=1}{\sum }}\omega_{i}K_{j}\left(X_{i}\right) \end{array}$$

where ω_*i*_ is the weight of the ith index.

6) Calculate the eigenvalue of the object to be evaluated and its class

If $K_{\text{j}0}(M)=\underset{\text{j}\in \left\{1,2,....,m\right\}}{\max}K_{j}(M_{x})$, then M is evaluated as class j0, with:


(18) $$\bar{K_{j}(M)}=\frac{K_{j}(M_{x})-\underset{1\leq j\leq m}{\text{min}}K_{j}(M_{x})}{\underset{1\leq j\leq m}{\max}K_{j}(M_{x})-\underset{1\leq j\leq m}{\min}K_{j}(M_{X})}$$

then the grade eigenvalue of M is j^*^


(19) $$\begin{array}{l} j^{*}=\frac{\overset{m}{\underset{j=1}{\sum }}j•\bar{K_{j}\left(M\right)}}{\overset{\text{m}}{\underset{j=1}{\sum }}\bar{K_{j}\left(M\right)}} \end{array}$$

7) Prediction of the eigenvalue of the object.

### ARIMA model

Autoregressive integrated moving average model (ARIMA) emerged mainly to solve the prediction problem of non-stationary time series. The ARIMA model is a combination of an auto-regressive moving average (ARMA) model and a differential model. The ARMA model is composed of an auto regressive (AR) model and a moving average (MA) model (22). The ARIMA model performs a d-order difference operation on the non-stationary time series to obtain a stationary series, which is then predicted by the ARMA (p, q) model. The mathematical expression of the ARMA model is as follows:


(20) $$\begin{array}{l} {\text{x}}_{t}=\sum_{i=1}^{p}\alpha_{i}x_{t-i}+u_{t}+\sum_{i=1}^{q}\theta_{i}u_{t-i} \end{array}$$

Where α_*i*_ is the autoregressive parameter, *u*_*t*_ is a white-noise process. The first part of the formula is the autoregressive process, and the second part is the moving average process. p, d, q represent the autoregressive order, the number of differences, and the moving average order, respectively.

The modeling steps of the ARIMA model are divided into the following points:

① Smoothness test of the time series and differencing of the unsteady data.
② Determination of the order by autocorrelation and partial autocorrelation tests, while the specific p and q values are determined by the AIC criterion.
③ White noise test of the residuals of the model, and *t*-test of the parameters.
④ Establishment of the model to select the appropriate interval for forecasting.

### Index selection

The APSC involves the production and circulation of agricultural products. It provides a channel to deliver products to the market before, during, and post-production in a smooth operation process, which is an important means to increase farmers' income and holds the key to ensuring food safety (23–25). The sustainable development concept emphasizes the coordinated development of nature, science and technology, economy, and society (26). To conduct in-depth research on the sustainable development of the APSC and to adapt to the long-term and efficient development of the supply chain, this paper adopts the analysis method to select indexes (27). By screening existing literature and referring to research and conclusions of related scholars, this paper selects a set of comprehensive, representative, and reasonable indexes (28) to reflect the nature of sustainable development of APSC. The chosen indexes are classified into technical conditions, ecological environment, economic and social, and infrastructure, as shown in Table 1. The paper also considers that the indexes selected should reflect reality and be accurate. The selected indexes and processed data are obtained from publicly available statistics in China.

**Table 1:** Indexes, and their definition and nature.

**Level I Index**	**Level II index**	**Definition**	**Nature**
Technical conditions A1	Rural generating capacity C1	Reflects the level of support for agricultural production	+
	Total power of agricultural machinery C2	Reflects production efficiency	+
	Level of device connectivity to the Internet C3	Measures the level of information-based life in rural areas	+
Ecological environment A2	Carbon emission of agricultural product logistics C4	Reflects the impact on the environment	-
Economic and social A3	Gross output value of farming, forestry, animal husbandry and fishery C5	Shows economic benefits produced	+
	Capital investment in agricultural product circulation C6	Measures the level of importance attached to agricultural product circulation	+
	Labor input in agricultural product circulation C7	Indicates the labor cost	+
Infrastructure A4	Quantity of comprehensive markets for agricultural products C8	Measures the intermediate sales level	+
	Total mileage of agricultural product transportation C9	Reflects transportation capacity	-
	Total nominal volume required for refrigeration C10	Indicates refrigeration volume required	-

### Data explanation

In terms of technical conditions, rural generating capacity and the total power of rural machinery both reflect the level of rural modernization, so they both play a positive role in promoting the sustainable development of APSC (29). The level of device connectivity to the Internet indicates the ratio of the number of users with rural broadband access to the number of rural households; it can better reflect the level of information-based rural life and facilitate the intelligent development of APSC (30).

In terms of ecological environment, the sustainable development of APSC necessarily requires an emphasis on environmental impacts; only green and low-carbon development is sustainable (31–33); this paper selects the widely-recognized index of carbon emission of agricultural product logistics as a negative index; this index is estimated with the energy consumption coefficient method proposed by IPCC. Multiply energies consumed in the circulation of agricultural products by the standard coal coefficient respectively and then multiply by the carbon emission coefficient to obtain the index. The formula can be referred to in the paper of Wang Xin (34).

On the economic and social fronts, the indexes of capital investment and labor input in agricultural product circulation are calculated by referring to Wang Renxiang's (35) method of multiplying by agricultural product circulation coefficient and the coefficient is measured by the final consumption rate multiplied by the proportion of household consumption in final consumption multiplied by Engel's coefficient. Many scholars have adopted this method to calculate data related to agricultural products.

In terms of infrastructure, comprehensive markets for agricultural products constitute a key part of the APSC. They are key places where various types of information gather, which will greatly improve the efficiency of agricultural product sales. In addition, the total mileage of agricultural product transportation can reflect the transportation efficiency and capacity of the APSC. The freight volume of railway, road, and waterway accounts for over 98% of the total freight volume in northeast China, then the total mileage of agricultural product transportation can be obtained by multiplying the sum of the lengths of the three routes by the coefficient of agricultural product circulation (36). With the rapid development of cold chain transportation and the increased quantity of fresh agricultural products, more emphasis should be put on the development of cold chain if the sustainable development of APSC is achieved (37); relevant data shows that cold chain transportation facilities in northeast China are yet to be improved; therefore, the total nominal volume required for refrigeration is selected in this paper as an index. The total nominal volume required for refrigeration reflects the demand for the infrastructure of fresh agricultural products, which can be calculated with formulas and volume utilization coefficient in the latest *Design Standards for Refrigeration*.

In conclusion, this paper selects data from Jilin, Liaoning, and Heilongjiang provinces in the period from 2007 to 2020 and adopts the entropy method and the matter-element tension model for analysis. Sources of the above index data include *China Population and Employment Statistics Yearbook, China Statistical Yearbook of the Tertiary Industry, China Agricultural Statistics, China Energy Statistical Yearbook, Jilin Statistical Yearbook, Liaoning Statistical Yearbook*, and *Heilongjiang Statistical Yearbook*.

## Results

### Classical domain and joint domain

The following panel data from 2007 to 2020 of the three northeast provinces in China are constructed according to the acquisition and calculation of the above indexes; the classical domain, determined by the improved index grading method, is divided into the following grades of basically sustainable, generally sustainable, comparatively sustainable, well sustainable:


$$\begin{array}{l} R_{1}\text{=}\left[\begin{array}{ccc} M_{1} & C_{1} & \left(30614.00,70745.25\right)\\ & C_{2} & \left(1678.30,2952.50\right)\\ & C_{3} & \left(0.05,0.17\right)\\ & C_{4} & \left(312.10,404.40\right)\\ & C_{5} & \left(1418.90,2673.70\right)\\ & C_{6} & \left(31.14,71.30\right)\\ & C_{7} & \left(3.87,7.97\right)\\ & C_{8} & \left(2.00,8.25\right)\\ & C_{9} & \left(19241.56,23356.07\right)\\ & C_{10} & \left(4844019.49,6144181.33\right) \end{array}\right]\\ R_{2}\text{=}\left[\begin{array}{ccc} M_{2} & C_{1} & \left(70745.25110876.50\right)\\ & C_{2} & \left(2952.504226.70\right)\\ & C_{3} & \left(0.170.28\right)\\ & C_{4} & \left(219.80312.10\right)\\ & C_{5} & \left(2673.703928.50\right)\\ & C_{6} & \left(71.30111.46\right)\\ & C_{7} & \left(7.9712.07\right)\\ & C_{8} & \left(8.2514.50\right)\\ & C_{9} & \left(15127.05,19241.56\right)\\ & C_{10} & \left(3543857.66,4844019.49\right) \end{array}\right]\\ R_{3}\text{=}\left[\begin{array}{ccc} M_{3} & C_{1} & \left(110876.50,151007.75\right)\\ & C_{2} & \left(4226.70,5500.90\right)\\ & C_{3} & \left(0.28,0.40\right)\\ & C_{4} & \left(127.49,219.80\right)\\ & C_{5} & \left(3928.50,5183.30\right)\\ & C_{6} & \left(111.46,151.62\right)\\ & C_{7} & \left(12.07,16.18\right)\\ & C_{8} & \left(14.50,20.75\right)\\ & C_{9} & \left(11012.55,15127.05\right)\\ & C_{10} & \left(2243695.82,3543857.66\right) \end{array}\right]\\ R_{4}\text{=}\left[\begin{array}{ccc} M_{4} & C_{1} & \left(151007.75,191139.00\right)\\ & C_{2} & \left(5500.90,6775.10\right)\\ & C_{3} & \left(0.40,0.52\right)\\ & C_{4} & \left(35.19,127.49\right)\\ & C_{5} & \left(5183.30,6438.10\right)\\ & C_{6} & \left(151.62,191.78\right)\\ & C_{7} & \left(16.18,20.28\right)\\ & C_{8} & \left(20.75,27.00\right)\\ & C_{9} & \left(6898.04,11012.55\right)\\ & C_{10} & \left(943533.99,2243695.82\right) \end{array}\right] \end{array}$$

Based on the above classical domains, the value range of the joint domain *R*_u_ is determined as follows:


$$R_{\text{u}}\text{=}\left[\begin{array}{ccc} M_{\text{u}} & C_{1} & \left(30614.00,191139.00\right)\\ & C_{2} & \left(1678.30,6775.10\right)\\ & C_{3} & \left(0.05,0.52\right)\\ & C_{4} & \left(35.19,404.40\right)\\ & C_{5} & \left(1418.90,6438.10\right)\\ & C_{6} & \left(31.14,191.78\right)\\ & C_{7} & \left(3.87,20.28\right)\\ & C_{8} & \left(2.00,27.00\right)\\ & C_{9} & \left(6898.04,23356.07\right)\\ & C_{10} & \left(943533.99,6144181.33\right) \end{array}\right]$$

### Index weight and correlation

This paper uses Stata 15 software to calculate the weight of each index by the entropy value method, as is shown in Table 2.

**Table 2:** Index weight.

	**C1**	**C2**	**C3**	**C4**	**C5**
Index weight	0.0684151	0.1265362	0.1291354	0.1051519	0.0910188
	**C6**	**C7**	**C8**	**C9**	**C10**
Index weight	0.1306379	0.1355876	0.0433803	0.1062997	0.0638371

It can be seen from the above table that the total power of agricultural machinery, level of device connectivity to the Internet, capital investment in agricultural product circulation, and labor input in agricultural product circulation has relatively higher weights, followed by the total mileage of agricultural product transportation and carbon emission of agricultural product logistics. It can be seen that in the process of sustainable development of APSC, human and financial resources provide the main support, and a fresh focus has been put on science and technology. In addition, the total mileage of transporting agricultural products and the amount of carbon emission generated during transportation are crucial to enhance transportation efficiency and environmental protection. Subsequently, the correlation of each grade from 2007 to 2020 in Jilin, Liaoning, and Heilongjiang provinces is calculated with the Matlab R2018b software, as is shown in Table 3.

**Table 3:** Correlation of the evaluation matter-elements in the three provinces.

	**Jilin**				**Liaoning**				**Heilongjiang**			
	**Grade 1**	**Grade 2**	**Grade 3**	**Grade 4**	**Grade 1**	**Grade 2**	**Grade 3**	**Grade 4**	**Grade 1**	**Grade 2**	**Grade 3**	**Grade 4**
2007	−0.160	−0.773	−0.691	−0.612	−0.192	−0.413	−0.328	−0.498	−0.101	−0.471	−0.704	−0.619
2008	−0.093	−0.690	−0.699	−0.599	−0.176	−0.262	−0.166	−0.485	−0.013	−0.394	−0.626	−0.615
2009	−0.023	−0.614	−0.652	−0.565	−0.195	−0.264	−0.298	−0.388	−0.118	−0.232	−0.585	−0.540
2010	−0.089	−0.606	−0.634	−0.487	−0.237	−0.223	−0.024	−0.426	−0.141	−0.204	−0.521	−0.496
2011	−0.028	−0.551	−0.600	−0.523	−0.230	−0.063	−0.141	−0.430	−0.119	−0.203	−0.484	−0.504
2012	−0.125	−0.537	−0.628	−0.425	−0.277	−0.130	−0.050	−0.401	−0.145	−0.272	−0.467	−0.486
2013	−0.168	−0.484	−0.614	−0.399	−0.334	−0.288	−0.253	−0.359	−0.173	−0.178	−0.234	−0.439
2014	−0.226	−0.483	−0.620	−0.446	−0.384	−0.213	−0.205	−0.383	−0.238	−0.250	−0.369	−0.411
2015	−0.272	−0.420	−0.573	−0.431	−0.304	−0.156	−0.083	−0.422	−0.296	−0.294	−0.227	−0.380
2016	−0.253	−0.449	−0.653	−0.393	−0.226	−0.192	−0.363	−0.479	−0.353	−0.296	−0.245	−0.343
2017	−0.265	−0.261	−0.485	−0.301	−0.181	−0.224	−0.291	−0.513	−0.444	−0.350	−0.265	−0.217
2018	−0.342	−0.408	−0.498	−0.240	−0.051	−0.112	−0.287	−0.555	−0.471	−0.363	−0.314	−0.118
2019	−0.305	−0.213	−0.496	−0.358	0.031	−0.212	−0.346	−0.604	−0.515	−0.469	−0.319	−0.143
2020	−0.267	−0.319	−0.532	−0.303	0.060	−0.303	−0.381	−0.609	−0.452	−0.373	−0.301	−0.281

### Grade variable eigenvalue and evaluation categories

Based on the above calculated weights and the correlation of each evaluation grade from 2007 to 2020 of the three provinces, the comprehensive correlation is calculated by using the Matlab R2018b software, then the grade variable eigenvalue is calculated based on the principle of maximum correlation, as is shown in Table 4.

**Table 4:** Grade variable eigenvalue.

	**Jilin**	**Liaoning**	**Heilongjiang**
2007	1.75547	1.75642	1.52799
2008	1.43408	2.01131	1.30824
2009	1.39543	1.74777	1.56263
2010	1.65140	2.26819	1.54497
2011	1.40091	2.10460	1.48331
2012	1.87867	2.30427	1.43758
2013	1.97967	2.40233	1.91690
2014	1.93373	2.51569	1.64956
2015	1.97426	2.30664	2.21337
2016	2.13874	1.79096	2.73636
2017	2.23609	1.86959	3.26540
2018	2.71421	1.80560	3.39443
2019	2.14024	1.70675	3.53108
2020	2.27193	1.63436	3.22977

**Figure 1:**
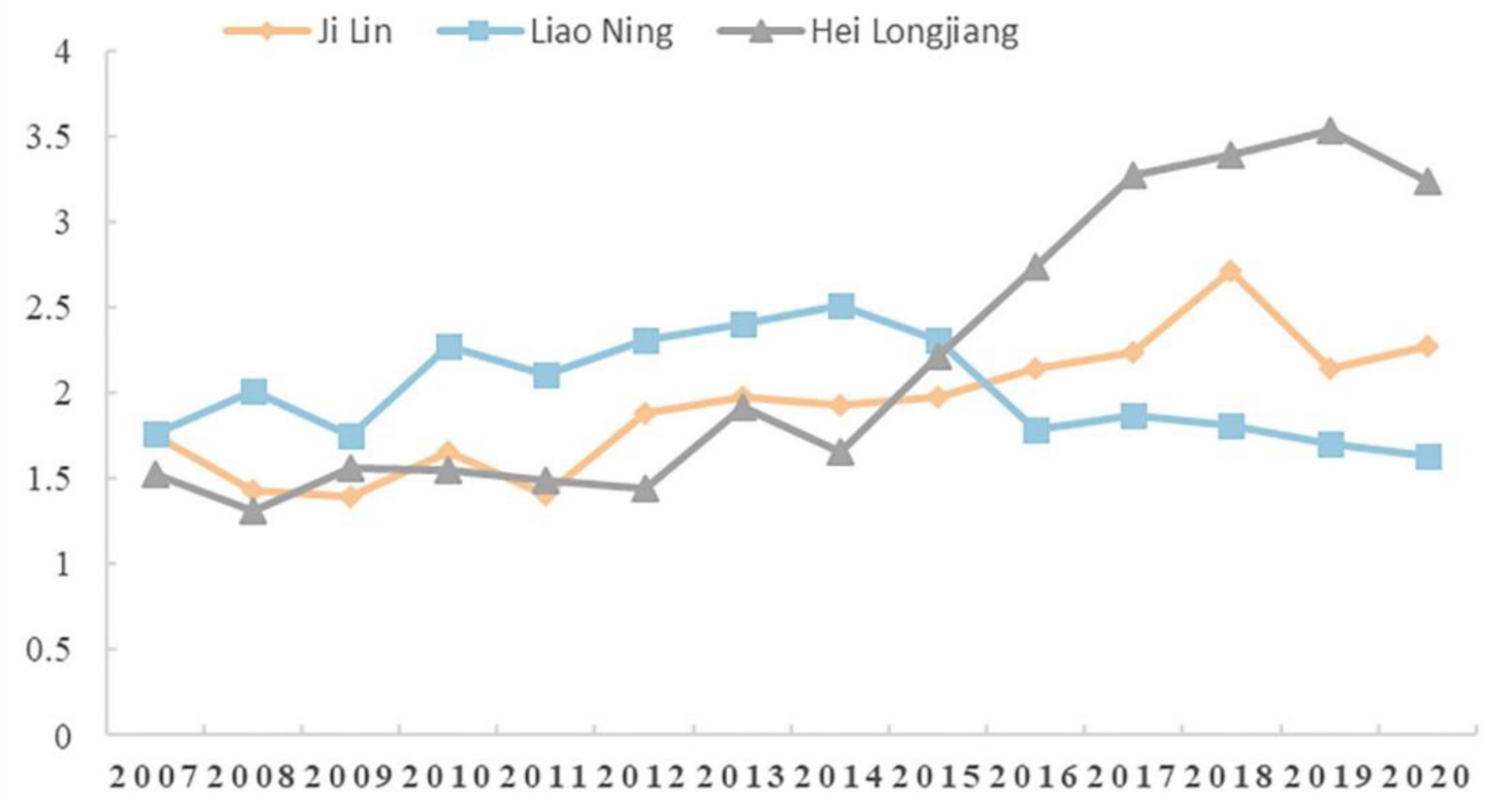
Line chart of evaluation grade changes of the sustainable development of APSC in the three provinces in Northeast China.

The eigenvalues of the sustainable development level variables of the APSC in the three provinces have changed to varying degrees from 2007 to 2020. To show the change more clearly, we made a graph, as shown in Figure 1.

We can visually see through Figure 1 that Jilin province as a whole shows a growing trend, basically consistent with the trend observed in Heilongjiang province from 2007-2014, and then grows slowly. 2007–2015, Liaoning province grade variable eigenvalue is greater than the other two provinces, and begins to decrease after 2015 shows and stabilizes at about 1.7. Heilongjiang province showed an overall up trend, flatter from 2007-2015, with rapid growth after 2014 and an inflection point in 2019, when it began to decline slightly, largely due to the impact of COVID-19 in 2020.

Corresponding evaluation categories can be obtained by analyzing the above grade variable eigenvalues. We have compiled the value, as shown in Table 5:

**Table 5:** Evaluation grade of sustainable development of APSC in the three northeast provinces.

	**Ji Lin**	**Liao Ning**	**Hei Longjiang**
2007	1	1	1
2008	1	3	1
2009	1	1	1
2010	1	3	1
2011	1	2	1
2012	1	3	1
2013	1	3	1
2014	1	3	1
2015	1	3	3
2016	1	2	3
2017	2	1	4
2018	4	1	4
2019	2	1	4
2020	1	1	4

The evaluation categories in Table 5 show the sustainability status of the APSC in the three provinces from 2007 to 2020. In Table 5, “1” indicates basically sustainable, “2” indicates generally sustainable, “3” indicates comparatively sustainable, and “4” indicates well sustainable. Except for 2017–2019, the APSC in Jilin province remains “basically sustainable,” but on the whole, it shows a slow upward trend. The APSC sustainability status of Liaoning province was significantly better than the other two provinces until 2015, but in recent years it has displayed a “basically sustainable” status and an undesirable development level. Similar to Jilin province, the APSC level in Heilongjiang province was maintained at “basically sustainable” until 2014, and after 2015, the sustainability level of the APSC in Heilongjiang province improved significantly and showed a strong growth trend, reaching and maintaining at a “well sustainable” status.

### Prediction of variable eigenvalues

Based on the above analysis, to further understand the sustainable development trend of the APSC in each province in the future, this paper uses the Autoregressive Integrated Moving Average (38) method to predict the time series of the eigenvalues of the level variables in the three northeast provinces from 2007 to 2020. This paper uses Anaconda software based on python 3.6.6 for analysis.

**Figure 2:**
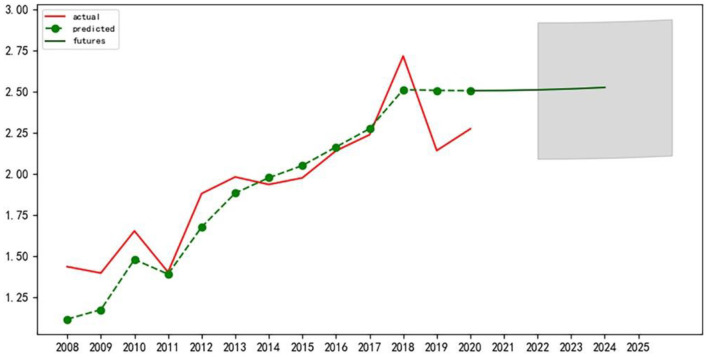
Prediction of variable eigenvalues of sustainable development level of APSC in Jilin Province.

**Figure 3:**
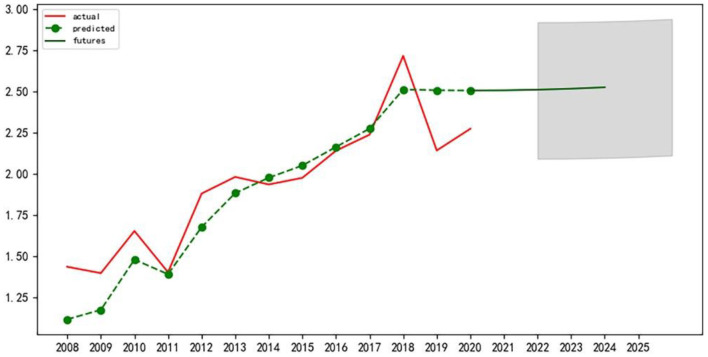
Prediction of variable eigenvalues of sustainable development level of APSC in Liaoning Province.

**Figure 4:**
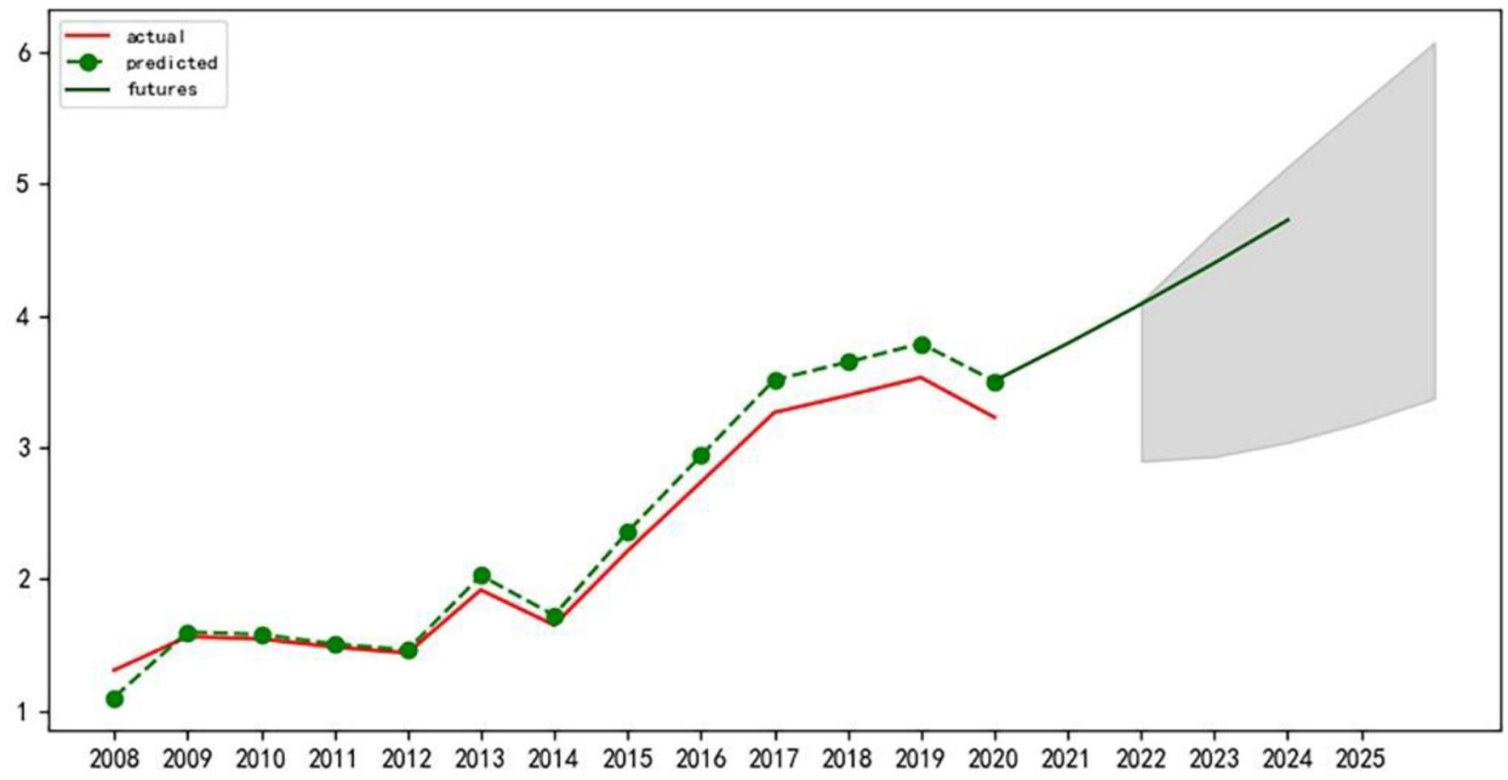
Prediction of variable eigenvalues of sustainable development level of APSC in Heilongjiang Province.

First of all, the author conducted the stationarity test of the time series data, and it is found that the data has strong volatility. After the test, this paper selects the second-order difference for processing to minimize data fluctuation and pass the relevant test. Next, the corresponding ARIMA model was constructed and tested for significance and normality of residuals and white noise tests, all of which passed the test. Finally, according to the Akaike information criterion, the model ARIMA (p, d, q) with the smallest AIC value is selected to predict and analyze the eigenvalues of the level variables of the sustainable development of the APSC in the three northeast provinces from 2021 to 2025 (39). Use the Matplotlib library for plotting, as shown in Figures 2–4 below:

We can see that the sustainable development of APSC in Jilin province will maintain a relatively stable upward trend in the next 5 years. The sustainable development of APSC in Liaoning province will be in a downward trend in the next 5 years, and a decrease of a relatively large margin will be recorded. The sustainable development of APSC in Heilongjiang province will show a rising trend in the next 5 years.

## Discussion

Based on the existing research, this paper proposes to study the sustainable development level of APSC from a macro perspective, which can provide better guidance for the sustainable development of the regional APSC. Based on the data related to the sustainable development of the APSC in the three northeast provinces from 2007 to 2020, we used the MEEM to obtain the level of sustainable development of the APSC in each province. Then ARIMA prediction is performed to obtain the sustainable development trend of APSC in each province in the next 5 years.

We found that the total power of agricultural machinery, level of device connectivity to the Internet, capital investment in agricultural product circulation, and labor input in agricultural product circulation occupy relatively high weights in the sustainable development of the APSC in the three northeast provinces, followed by the total mileage of agricultural product transportation and carbon emission of agricultural product logistics. Therefore, the three northeast provinces should put more emphasis on building a modern circulation system for agricultural products by fully harnessing the power of science and technology. Given the status quo of brain drain, more efforts should be made to enhance the attractiveness of human and financial resources. In this way, we can realize the smooth flow of agricultural products circulation process, reduce carbon emissions of agricultural products transportation, increase farmers' income, guarantee food quality and security, and make an important contribution to maintaining public health and wellness.

In addition, the sustainable state of the APSC in Jilin province has been gradually improved in recent years and maintained a slowly growing trend. The sustainable development of the APSC in Liaoning province showed strong momentum several years ago but has declined significantly in recent years, pointing to a bigger decrease in the future. The APSC in Heilongjiang province is in a good state of sustainability and shows a tendency for marked growth. As such, Jilin province should take bold steps in innovation while maintaining the status quo, and work on scientific and technological fronts to fully utilize its resources. Liaoning province, with much room for improvement, can draw inspiration from Heilongjiang province. Liaoning province needs to solve the issue of resource mismatch as soon as possible and focus more on the coordination of the overall operation of the APSC. All participants need to increase the importance of APSC and make the timely adjustment to relevant strategies to prevent the weakening of the sustainable development capacity of the APSC. Heilongjiang province should set a benchmark for the sustainable development of the APSC, strengthen regional cooperation and sharing, and amplify positive influences.

The research results obtained in this paper can be verified in the research of other scholars. Dainan Hou concluded that Heilongjiang, Jilin, and Liaoning provinces should be placed in descending order in exploring the relationship between agriculture and environmental friendliness in the three northeast provinces (27). Chen Xu's analysis of indexes in the Rural Vitalization Plan of Jilin Province (2018–2022) indicates that Jilin province is endowed with abundant natural resources, but without being fully utilized. The improvement of agricultural machinery driven by the implementation of the rural vitalization strategy represents the increased total power of agricultural machinery and more efficient production at the source of the APSC; planning is also underway for the agricultural product circulation system, which has a promising prospect (40). In addition, according to the research of Zhang Xue et al., the agricultural infrastructure in Liaoning province is fully-fledged, but matching resources are not put in place in recent years. The migration of young and middle-aged labor force to cities has led to the shrink of large-scale agricultural operations and the development of industries with local advantages. Meanwhile, it restricts the sustainable development of APSC, putting a bottleneck on the sustainable development of the APSC of Liaoning province (41, 42). At the same time, according to the research of the Heilongjiang Agricultural and Rural Development Research Center and Heilongjiang Academy of Agricultural Sciences on the implementation of the rural vitalization strategy, Heilongjiang province attaches great importance to the sustainable development of the APSC. It's gaining momentum in the digitalization drive by integrating upstream and downstream systems in the supply chain and connecting the sales of key agricultural products to the Internet. A provincial e-commerce platform for agricultural products is gradually taking shape. Meanwhile, Heilongjiang does well in cultivating the labor force capable of engaging in the circulation of agricultural products (43, 44).

Through the above policy research and analysis, it can be seen that the research results of some scholars on the sustainable development of agriculture and APSC in the three northeast provinces are consistent with the data analysis results and development trends of some indicators in this paper. This not only demonstrates the authenticity and accuracy of the research in this paper but also enhances the credibility of innovative research conducted in this paper. In addition, the analysis from a systematic perspective in this paper complements and connects the above studies, making up for the data deficiencies in policy analysis and management suggestions from empirical research.

The combined use of the MEEM and ARIMA prediction model is pioneering in this type of research, which can be replicated in other areas of research and advance the development of evaluation type of research. We can not only accurately obtain the weights of various influencing factors, but also increase the objectivity of the evaluation results of sustainable development of the APSC in the three northeast provinces and make it more consistent with the actual situation. The forecast section takes into account factors such as random effects, allowing for more accurate short-term forecasts and reliable results.

## Conclusion

In summary, this study fills the research gap in the sustainable development of the APSC in three northeast provinces of China. In terms of modeling methods, the MEEM and ARIMA prediction model are used for the first time, which is proved to be innovative and transferable. The findings of this paper can provide useful references for governments, enterprises, and different stakeholders in the three provinces, improve policymakers' understanding of the value of the APSC, and can be applied to the governance of the APSC in other areas tackling the problem of unbalanced development. In practice, the assessment also guides the quality and safety of agricultural products and public health and is important for the sustainable development of agroecology and the environment. However, there are still limitations on the research sample in this paper. Although the three northeast provinces are three important regions for agricultural supply in China, in the future, the sample scope can be increased to study the sustainable development of the APSC at the national level to expand comparability. In addition, we can also analyze the specific influencing factors in detail and simulate the sustainable development paths, so as to solve the sustainable development problem of APSC in different countries.

## Data availability statement

Publicly available datasets were analyzed in this study. This data can be found here: https://data.stats.gov.cn/, https://data.cnki.net/yearbook/Single/N2021050066, https://data.cnki.net/yearbook/Single/N2020120488, http://tjj.jl.gov.cn/tjsj/tjnj/, http://tjj.ln.gov.cn/tjsj/sjcx/ndsj/, and http://tjj.hlj.gov.cn/tjsj/tjnj/.

## Author contributions

Conceptualization and funding acquisition: XF. Methodology, software, resources, writing—review, and editing: XF and YZ. Validation: CZ, BL, and YZ. Formal analysis: BL, HC, and YM. Data curation: YM, BL, and CZ. Writing—original draft preparation: XF, YZ, YM, and CZ. Visualization: YZ and YM. Supervision and project administration: XF and HC. All authors have read and agreed to the published version of the manuscript.
